# Self-Medication among Medical and Nonmedical Students at the University of Gondar, Northwest Ethiopia: A Cross-Sectional Study

**DOI:** 10.1155/2020/4021586

**Published:** 2020-06-12

**Authors:** Zelalem T. Tesfaye, Asrat E. Ergena, Bilal T. Yimer

**Affiliations:** ^1^Department of Pharmacology and Clinical Pharmacy, School of Pharmacy, College of Health Sciences, Addis Ababa University, Addis Ababa, Ethiopia; ^2^Department of Pharmaceutical Chemistry, School of Pharmacy, College of Medicine and Health Sciences, University of Gondar, Gondar, Ethiopia; ^3^Department Pharmaceutics, School of Pharmacy, College of Medicine and Health Sciences, University of Gondar, Gondar, Ethiopia

## Abstract

Despite having some benefits, self-medication increases risks such as unnecessary use of medication, extended duration of consumption, incorrect diagnosis, drug-drug interactions, and polypharmacy. Thus, the purpose of this study is to compare self-medication practice between medical and nonmedical students of the University of Gondar, Northwest Ethiopia. An institutional-based cross-sectional comparative study was conducted on medical and nonmedical students of the University of Gondar from March 25 to May 15, 2018. A comparative sample of 213 medical and 212 nonmedical students were enrolled in the study. Data were collected by physically visiting the students in their campuses, using a semistructured questionnaire. Of the participants with a history of medication use in the past 12 months, 64.5% practiced self-medication. The prevalence of self-medication was 59.7% among medical students and 69.0% among nonmedical students. “Knowing the treatment of the disease” was the most frequent reason behind self-medication. Analgesics/antipyretics were the most common categories of medications used, whereas headache was the predominant ailment for which the medications were used. Self-medication practice was found to be higher in the fifth year students and nonmedical students (*p* < 0.05). In conclusion, self-medication is common among students of the University of Gondar. Nonmedical students were more likely to have practiced self-medication as compared to medical students.

## 1. Introduction

Self-medication is the selection and usage of medications by individuals to treat self-identified illnesses or symptoms. It is otherwise defined as the use of nonprescription drugs by people on their own initiative [1]. When practiced appropriately, self-medication could have positive impact on the individual and the healthcare system by potentially saving lives in acute conditions, saving the time spent in waiting for a clinician, and contributing to decrease in the healthcare cost. On the contrary, self-medication increases risks such as unnecessary use of medication, extended duration of consumption, incorrect diagnosis, drug-drug interactions, and polypharmacy [2, 3].

Studies show that college students have very low tendency to consult health professionals to seek health-related information, to get treatment, or to obtain other healthcare services [4]. In the recent years of increased social media influence, students rely more on the Internet for information regarding their health rather than consulting healthcare professionals [5, 6]. This increases the likelihood of practicing self-medication among college students to treat self-diagnosed illnesses [7].

Previous studies have shown that medical knowledge is one of the factors that could have an impact on self-medication practice among college students [8, 9]. With the assumption difference in the level of preexisting medical knowledge, several studies have compared self-medication between medical and nonmedical students [10–12]. However, it is difficult to establish a clear relationship between the role of medical education and self-medication practice among students because of divergent results of the studies.

Few studies have been issued previously on self-medication practices of Ethiopian college students [13–17]. However, due to the fast-changing sociocultural environment in Ethiopian universities, it is important to analyse the contemporary status of self-medication practice among students. Moreover, comparing the extent of self-medication between medical and nonmedical students is important to identify potential difference in self-medication practice between the groups as well as to provide baseline information for researchers and public health officials who work on the subject. Thus, the purpose of this study is to compare self-medication practice among medical and nonmedical students of the University of Gondar, Northwest Ethiopia.

## 2. Materials and Methods

An institutional-based cross-sectional comparative study was conducted on medical and nonmedical students of the University of Gondar from March 25 to May 15, 2018. Sample size (N) was calculated using the following formula:

*N*=*Z*_*α*/2_^2^ × *P*(1 − *P*) /*δ*^2^ [18], where the proportion (*p*) of self-medication among the population is assumed to be 50%. Taking a *Z* value for 95% confidence interval (CI) (1.96) and margin of error (*δ*) of 5%, the estimated sample size was calculated to be 384. For contingency, 10% of the estimation was added to the sample, producing the final sample size of 425.

Students from the College of Medicine and Health Sciences, University of Gondar (213 students), were enrolled as medical students, whereas students from the College of Natural and Computational Sciences, University of Gondar (212 students), were enrolled as nonmedical students. Since data available on the source population were insufficient to generate the sampling frame, the convenience sampling method was used to select study participants.

A semistructured questionnaire was used to collect the data. The questionnaire was prepared from similar studies conducted previously in Ethiopia [13, 15–17] by adapting to the study purpose and population. The questionnaire consisted of two sections: sociodemographic characteristics and practice of self-medication. Data were collected by physically visiting the students in their campuses. Data were checked, cleaned, and entered into IBM SPSS Statistics^®^ Version 20 for analysis. Results were described using percentage, mean, and standard deviation (SD). Frequency tables and graphs were used to summarize the findings.

Furthermore, univariate and multivariate logistic analyses were carried out to determine the predictors of self-medication practice among students. Logistic regression analysis is applied for testing hypotheses about relationships between a categorical outcome variable and one (univariate) or more (multivariate) categorical or continuous predictor variables [19]. A *p* value < 0.05 was set as an indicator of significant relationship.

Ethical clearance was obtained from the Ethical Review Board of the School of Pharmacy, University of Gondar. Written consent was acquired from the respondents for enrolment in the study. Besides, confidentiality of the information was strictly maintained throughout data collection and data analysis process.

## 3. Results

### 3.1. Sociodemographic Characteristics

Of the 425 participants enrolled, 59.1% were males. The participants' age ranged from 21 to 30 with a mean age of 21.35± 1.52. Third year students accounted for 42.1% of the participants (Table 1).

### 3.2. Assessment of Practice of Self-Medication

**Figure 1:**
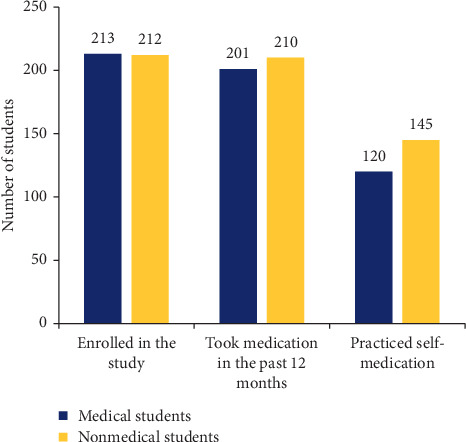
Prevalence of self-medication practice among medical and nonmedical students.

Of the total 425 students who participated in the study, 411 (96.7%) reported that they had taken medication in the preceding 12 months. Twelve medical students and two nonmedical students did not recall taking medications during the same period. Of those with the history of medication use, 265 (64.5%) took medication without prescription (practiced self-medication). The prevalence of self-medication was found to be higher among nonmedical students (Figure 1).

The students were asked to describe different aspects of their most recent self-medication practice. As a result, “knowing the treatment of the disease” was the most frequent reason (39.6%) for self-medication. Analgesics/antipyretics were the most common categories (58.5%) of medications used, whereas headache was the predominant ailment (42.7%) for which the medications were used (Table 2).

### 3.3. Factors Associated with Self-Medication

Binary logistic analysis was performed to identify predictors for self-medication practice. Age and sex did not show significant association with self-medication in both univariate and multivariate analyses. On the contrary, the fifth year students [AOR: 5.524 (1.436, 21.244)] were more likely to practice self-medication. When we compared medical students to nonmedical students, the odds of self-medication practice were found to be lower among medical students [AOR: 0.375 (0.155, 0.907)] (Table 3).

## 4. Discussion

Self-medication can be useful or harmful for an individual's health, depending on factors such as the medication used, type of illness, source of the medication, and the practiser's level of knowledge [20]. There could be a difference between medical and nonmedical students with respect to the level of awareness on issues related to self-medication. Thus, the implication of self-medication in medical students would not be the same as that of nonmedical students [11].

In this study, the overall rate of self-medication was 64.5% among students who had taken medications in the preceding twelve months. Other studies conducted on Ethiopian universities reported self-medication rates lower than our finding [13, 15, 16]. However, it was difficult to compare our finding with these reports because their report covered students' practices over less than a six-month period, whereas our finding represented students' practices over a 12-month period. The prevalence of self-medication among our study population was found to be lower than studies conducted on university students in Nigeria (81.8%) [10], in Brazil (86.4%) [21], in Slovenia (92.3%) [11], and in Jordan (96%) [22]. Different factors such as accessibility of medications, access to primary healthcare service, medication-related regulations, and students' health-seeking behaviour differences across the study settings may be responsible for the difference in the prevalence of self-medications. For instance, university students in our study have access to free healthcare service from the university which may encourage students to visit student clinics instead of practicing self-medication. Although the prevalence of self-medication was lower than several studies from elsewhere, the practice of self-medication in our setting is significant.

The predominant ailment that led to self-medication in our study was headache (42.7%), followed by cough and cold (21.1%). This finding agrees with the findings of other studies conducted at three universities in Ethiopia, all of which reported that headache and cough/common cold were among the most common ailments that led to self-medication [13, 15, 16]. This similarity indicates university students regard headache, cough, and cold as ailments that do not need medical checkup. Although it is possible to get symptomatic relief from over-the-counter medications, these ailments may develop as symptoms of more serious underlying disease. Thus, it is important to create awareness among university students on the importance of consulting health professionals even if they consider their symptoms to be minor.

Regarding the types of medications used for self-medication, analgesics were used in more than half of the cases (58.5%), followed by antibiotics (26.5%). Similarly, analgesics and antibiotics were found to be the two most common types of medications in other studies conducted in Ethiopia [13, 15, 16] and Nigeria [10], while a Palestinian study reported analgesics to be the most commonly used and antibiotics to be in the third place, following decongestants [12]. The similarity observed may be due to comparable economic status, and hence comparable level of development of healthcare system among these countries. The frequent use of analgesics is fairly understandable as there are many over-the-counter analgesic medications intended for mild-to-moderate pain relief. In contrary, the use of antibiotics in self-medication should be strictly prohibited because it will facilitate the development of antimicrobial-resistant strains, which has become a major challenge to public health globally.

In the present study, age and sex of the students did not show effect on their self-medication practice. Other studies also reported the absence of significant association between self-medication and age [16, 21, 23] as well as self-medication and sex [12, 23]. In contrast with our finding, several studies reported that female students were more likely to practice self-medication [10, 16, 21, 24]. Self-medication related to premenstrual symptoms may contribute to a higher rate of female self-medication. The fact that dysmenorrhea-related self-medication accounts for only 3.4% of the cases in our study might be the reason behind similarity of the likelihood of practicing self-medication between male and female students. Regarding the year of study, the fifth year students were found to be more likely to have practiced self-medication. In accordance with this, a study conducted in a university in Nigeria found out that senior students were more likely to practice self-medication [10]. Various psychosocial changes that develop with campus life could contribute to difference in the practice of self-medications across different levels of students' seniority.

In this study, the odds of medical students practicing self-medication were lower than nonmedical students. In contrary to this finding, a Nigerian study on university students reported higher odds of self-medication among medical students [10], while several other studies found no significant difference in self-medication between medical and nonmedical students [11, 21, 23]. Higher likelihood of nonmedical students in our study setting to practice self-medication could be explained by lesser convenience for visiting primary care facilities. Medical students reside in a campus where the university hospital is located, whereas nonmedical students reside further across the town, making self-medication more convenient than visiting the hospital.

The limitations of the study should not be disregarded. First of all, the study did not address difference in self-medication across students of different universities since study subjects were enrolled from one university. In addition, as the study employed a cross-sectional study design, it is not possible to establish cause and effect relationship between independent and dependent variables. Therefore, the findings of this study should be interpreted by taking its limitations into account.

## 5. Conclusion

Our study revealed that self-medication is common among students of the University of Gondar. Headache was the most common ailment that led to self-medication, whereas analgesics were the most commonly used class of medications for self-medication. Nonmedical students were more likely to have practiced self-medication as compared to medical students.

## Figures and Tables

**Table 1:** Sociodemographic and academical background of participants.

Variables	Frequency (%)
*Sex*	
Male	247 (58.1)
Female	178 (41.9)

*Age in years*	
18–21	242 (56.9)
22–25	182 (42.8)
>25	1 (0.2)

*Faculty*	
Medical	213 (50.1)
Nonmedical	212 (49.9)

*Year of study*	
First year	44 (10.4)
Second year	134 (31.5)
Third year	179 (42.1)
Fourth year	33 (7.8)
Fifth year	35 (8.2)

*Monthly income in ETB*	
100–500	311 (72)
501–1000	90 (21.2)
1001–1500	18 (4.2)
1501–2000	6 (1.4)

*Place of residence*	
Campus dormitory	423 (99.5)
Nondormitory	2 (0.5)

Abbreviation: ETB, Ethiopian birr (Ethiopian birr/US dollar = 0.037 during the study).

**Table 2:** Associated reasons, illnesses, and common medications for the most recent self-medication.

Variable	Frequency (%)
*Reason for self-medication*	
Shortage of money	52 (19.6)
Shortage of time	13 (4.9)
Do not like visiting and getting checked up	27 (10.2)
Mildness of illness	65 (24.5)
An already known disease	105 (39.6)
Other reasons	3 (1.1)

*Sources of drugs for self-medication*	
Private pharmacies	218 (82.2)
Governmental pharmacies	24 (9.1)
Left-over medications from previous use	11 (4.2)
Friends/roommates	12 (4.4)

*Conditions led to self-medication*	
Headache	113 (42.7)
Cough and common cold	56 (21.1)
Pain (epigastric, tooth, body)	57 (21.1)
Diarrhea	16 (6.1)
Dysmenorrhea	9 (3.4)
Other medical conditions^*∗*^	14 (5.3)

*Categories of medications used*	
Analgesics	155 (58.5)
Antacids	10 (3.8)
Antispasmodics	24 (9.1)
Antibiotics	70 (26.5)
Others	6 (2.3)

^*∗*^Others: dyspepsia (8), tonsillitis (3), and skin problems (2).

**Table 3:** Factors associated with self-medication practice.

Variables	Self-medication		Odds ratio (95% confidence interval)		*p* value^*∗*^
	Yes	No	Crude	Adjusted	
*Sex*					
Male	158	78	1.287 (0.857, 1.935)	1.102 (0.637, 1.909)	0.728
Female	107	68	1.00	1.00	

*Faculty*					8
Medical	120	81	**0.664 (0.442, 0.997)**	**0.375 (0.155, 0.907)**	**0.030 ** ^*∗∗*^
Nonmedical	145	65	1.00	1.00	

*Year of study*					
First year	31	13	1.00	1.00	
Second year	78	51	0.641 (0.307, 1.314)	0.835 (0.345, 2.020)	0.689
Third year	106	67	0.663 (0.324, 1.358)	1.028 (0.393, 2.686)	0.956 0
Fourth year	22	11	0.839 (0.318, 2.215)	2.120 (0.665, 6.766)	0.204
Fifth year	28	4	2.935 (0.857, 10.060)	**5.524 (1.436, 21.244)**	**0.013 ** ^*∗∗*^

Notes: ^*∗*^*p* values characterize the corresponding adjusted odds ratios. Bold values indicate significant predictors at 95% confidence level. ^*∗∗*^Significant value (*p* < 0.05).

## Data Availability

The SPSS dataset used to support the findings of this study is available from the corresponding author upon request.
